# Assessing the Burden of Thromboembolic Complications in Outpatient COVID-19 Cases: A Focused Study on Patients Followed in Primary Care

**DOI:** 10.1155/cjid/7242450

**Published:** 2025-07-24

**Authors:** Buğu Usanma Koban, Özla Çelik, Ülkü Sur Ünal, Arzu Uzuner

**Affiliations:** ^1^Family Medicine Department, Marmara University School of Medicine, İstanbul, Turkey; ^2^Family Medicine, Fenerbahçe Family Healthcare Center, İstanbul, Turkey

**Keywords:** COVID-19, deep vein thrombosis, outpatient, primary care, pulmonary embolism, thromboembolic event

## Abstract

**Background:** Thromboembolic complications are frequently observed in patients with COVID-19 infection, particularly among those admitted to the intensive care unit (ICU). Moreover, COVID-19 infections today are mostly characterized by mild symptoms and are managed primarily in primary care settings, resulting in limited literature data on thromboembolic complications in this patient group. This study aims to investigate thrombotic event risks and associated factors in the outpatient population.

**Methods:** All outpatient COVID-19 cases managed at two family health centers in Istanbul between June 2020 and December 2021 were retrospectively reviewed. Patients were contacted and informed about the study. Sociodemographic and clinical data were obtained from family health center records, and information on thrombotic events was extracted from hospital discharge summaries. Fisher's exact test and logistic regression were used for analysis.

**Results:** A total of 961 patients were included in the study, of whom, 519 (54%) were female and 442 (46%) were male. The mean age was 41 years (range: 18–97). Thromboembolic events occurred in 4 patients (0.42%) within the first 6 months following COVID-19 infection. Logistic regression analysis identified the use of antacid medications (*p*=0.01) and the presence of hematological disorders (*p*=0.03) as significant risk factors.

**Conclusion:** Thromboembolic events may occur even in mild-to-moderate outpatient COVID-19 cases within six months following the infection. Risk assessments focusing on comorbidities and medication use should be performed during outpatient follow-up. Due to the small number of thromboembolic events in this study, the findings should be considered preliminary and interpreted with caution.

**Trial Registration:** ClinicalTrials.gov identifier: NCT06695377

## 1. Introduction

COVID-19, caused by the severe acute respiratory syndrome coronavirus 2 (SARS-CoV-2), was first identified in Wuhan, China, in 2019 and rapidly escalated into a global pandemic with high morbidity and mortality rates. According to current data, COVID-19 has resulted in approximately 776 million cases and 7.1 million deaths worldwide, while in Turkey, it has caused 17.2 million cases and 102,000 deaths [1, 2].

SARS-CoV-2 exhibits a wide clinical spectrum, ranging from asymptomatic infection to fatal outcomes. As the name suggests, it is primarily a respiratory disease that can lead to pneumonia or acute respiratory distress syndrome (ARDS) in severe cases [3]. According to the literature, 10%–20% of cases require hospitalization, and 2%–4% necessitate treatment in intensive care units (ICUs) [4]. In addition to respiratory symptoms, nonrespiratory manifestations, including venous and arterial thromboembolic events, have been reported in COVID-19 patients. Various groups have documented thromboembolic complications in these patients [5–8]. Significantly, vascular structures of multiple organs, including the lungs, legs, spleen, heart, and brain, are affected by COVID-19-induced coagulopathy [5–7, 9–13]. These complications are particularly associated with multiorgan failure and higher mortality rates in critically ill patients [14]. Pulmonary embolism (PE) and deep vein thrombosis (DVT) have been identified as the most prevalent thrombotic events associated with COVID-19 [8]. However, data on the occurrence of these events in nonhospitalized patients with mild-to-moderate symptoms remain scarce, and the necessity for prophylactic interventions in such cases is not well established. From a pathophysiological perspective, SARS-CoV-2 belongs to the same family as the original SARS virus and uses the angiotensin-converting enzyme 2 (ACE2) receptor for cell entry [15]. Since ACE2 is also expressed in myocardial tissue, this provides a potential mechanistic link between COVID-19 and cardiovascular complications. Experimental studies in mice, as well as in human autopsy reports, have demonstrated that COVID-19 downregulates ACE2 expression in both cardiac and pulmonary tissues, which may contribute to myocarditis. In addition, the infection triggers elevated levels of proinflammatory cytokines across multiple organs, increasing the likelihood of multiorgan damage in severe cases [16]. The procoagulant effects of systemic infection due to viral infection increase the risk of thrombosis, making intensive anticoagulant therapy particularly necessary for individuals with a history of cardiovascular events [17].

Despite growing concerns, short-term cardiovascular outcomes following COVID-19 infection remain insufficiently studied. A study conducted by the World Heart Federation, involving 5313 patients from 23 countries, reported cardiac arrest in 5.5%, heart failure in 3.8%, and myocardial infarction in 1.6% of hospitalized COVID-19 patients, with a 20% rate of sudden cardiac death [18]. Similarly, a Danish cohort study examining cardiovascular events within 180 days post-COVID-19 infection found evidence supporting an increased cardiovascular risk associated with the infection [19]. In the United States, large vessel occlusion and stroke were observed in five patients under the age of 50 [20]. However, data from Turkey on this subject are insufficient. Regarding nonhospitalized COVID-19 cases, knowledge is even more limited, as most studies have focused on hospitalized patients.

Although COVID-19 infections continue worldwide, largely due to the impact of vaccination, they are mostly experienced with mild symptoms. In this context, it becomes increasingly important for primary care physicians, who often serve as the first and only point of contact for these patients, to understand the potential for thromboembolic complications. Identifying the incidence and potential risk factors in this population is crucial for clinical decision-making. Therefore, the primary aim of our study is to investigate the frequency and associated causes of cardiovascular events, cerebrovascular events, and venous thrombotic events within the first six months following COVID-19 infection in patients followed in primary care. By focusing on mild-to-moderate cases, we seek to contribute to the literature regarding the circulatory system effects of COVID-19 infection in outpatient settings.

## 2. Materials and Methods

This study included patients aged 18 years and older who were registered at family health centers in two different districts of Istanbul and had confirmed COVID-19 infection verified by PCR testing between January 1, 2022, and June 30, 2022. A total of 1040 patients meeting these criteria were identified from family health center records, and it was aimed to reach the entire population. Patients were contacted via telephone, and those not reached on the first attempt were called up to three times over three consecutive days. After being informed about the study, 961 patients agreed to participate and completed a 12-item questionnaire designed by the researchers to collect sociodemographic and medical history data. Participants were asked about their chronic illnesses, medications used, date of COVID-19 infection, treatments received for COVID-19, and whether they experienced any cardiovascular or cerebrovascular events, PE, DVT, or other circulatory pathologies within the first 6 months following the infection. This information was cross-verified with family health center records. Data on thromboembolic events were obtained from discharge summaries available in the national medical record system. The medications used by patients were categorized based on the Anatomical Therapeutic Chemical (ATC) Classification System.

The data were analyzed using SPSS software (Version 20.0, SPSS Inc., Chicago, IL, USA). Categorical variables were analyzed using the chi-square and Fisher's exact tests. Variables showing statistically significant associations were further examined using logistic regression analysis. A *p* value of < 0.05 was considered statistically significant.

## 3. Results

Among the 961 patients included in the study, 519 (54%) were female, and 442 (46%) were male. The mean age was 41 years, with a minimum age of 18 and a maximum age of 97. Thromboembolic events occurred in 4 patients (0.42%) within the first 6 months following COVID-19 infection. The sociodemographic and medical characteristics of the participants are summarized in Table 1.

A comparative analysis of the characteristics of patients who experienced thromboembolic events and those who did not revealed that individuals with hematological disorders and those receiving antithrombotic therapy had a higher risk of thrombosis (*p* < 0.05). No statistically significant associations were found between thromboembolic events and gender, age, other comorbidities, medications used, or treatments received for COVID-19.

Logistic regression analysis showed that the use of antacid medications (classified under the A02 drug group) and the presence of hematologic disorders were independently associated with an increased risk of thromboembolic events (Table 2).

Of the four participants who experienced thromboembolic complications, all were female, with ages ranging from 25 to 75 years. Among them, two had DVT, one had PE, and one had myocardial infarction. The characteristics of these patients are presented in Table 3.

## 4. Discussion

In this study, thromboembolic complications were observed in 4 out of 961 COVID-19 patients followed in primary care within the first 6 months postinfection. The risk of thromboembolism appeared to be elevated in individuals using antacid medications and in patients with underlying hematologic diseases.

Reported rates of thromboembolic events following COVID-19 infection vary across the literature. Studies focusing on patients treated in ICUs have shown venous thromboembolism (VTE) incidence rates ranging from 23.3% to 30%, while rates in hospitalized patients overall fall between 14% and 17% [21, 22] However, these frequencies are mostly calculated in patients with a history of hospitalization, including those in ICUs. In contrast, a study conducted among patients managed in primary care found an incidence of 0.9% for DVT or superficial vein thrombosis among 8547 COVID-19 cases over a one-year period [23] In our study, among 961 patients followed by family physicians at home with mild-to-moderate symptoms, 4 patients were found to have experienced thrombotic events (0.42%). While the participants in the referenced study were screened between 2020 and 2021, our study included patients who tested positive via PCR as of January 2022. The difference in complication rates could potentially be attributed to factors such as the predominance of vaccinated individuals and the relative scarcity of factors that increase cardiovascular risks, such as reduced physical activity due to strict lockdown measures being relaxed.

Similarly, in a retrospective study of hospitalized patients, it was observed that the majority of thromboembolic events involved PE and DVT [24]. In addition, in a large-scale Swedish study that compared 1,057,174 SARS-CoV-2-positive individuals with controls, the incidence of DVT and PE increased significantly within 70 and 110 days postinfection, respectively. These risks were particularly higher in critically ill patients and those infected during the first wave of the pandemic [25]. Based on this information, we can see that the period with an increased incidence after infection is covered in our follow-up of patients over 6 months. This is important for minimizing the potential underdetection of relevant cases. Several pathophysiological processes are believed to contribute to thromboembolic events in COVID-19 patients. The most prominent among these are systemic inflammation, immobility, and a prothrombotic environment [26]. The mechanism of prothrombosis is quite complex. Hyperactive complement and coagulation systems, endothelial dysfunction, cytokine storm, and platelet activation contribute to this environment [27–29]. Thus, immunothrombosis and thromboinflammation are likely to act synergistically. In this regard, understanding these processes is essential for evaluating thrombotic risk, even in nonhospitalized patients. Immunological triggers can be monitored via laboratory markers, one of which is the elevation of antiphospholipid antibodies, a phenomenon reported in 46.8%–52% of hospitalized COVID-19 patients [30, 31] In our study, elevated antiphospholipid antibodies were detected in one of the patients who experienced a thrombotic event. Although this observation is limited to a single case, it raises the possibility that such antibody elevations may occur transiently, even in mild cases. Further research is warranted to explore this association. In reviewing patients' general health status and comorbidities, it becomes evident that the baseline blood values, which change during the infection process, and post-COVID-19 follow-up of patients with hematological diseases are crucial [26]. In this study, one of the patients who experienced a thrombotic event had a history of hematological disease. While this finding is consistent with existing literature, the small number of events limits the strength of this association. No significant association was found between thrombotic risk and any other comorbidity.

Our regression analysis also indicated a potential link between the use of antacid medications and an increased risk of thromboembolism. However, this observation should be interpreted with caution due to the very small number of events and potential confounding factors. A thorough literature review did not identify prior evidence supporting such an association, which suggests that the finding may be incidental. Moreover, antacids are commonly prescribed to patients with chronic conditions who are also receiving other medications, including anticoagulants. Therefore, it is difficult to determine whether the observed association reflects a true pharmacological effect or is a result of underlying comorbidities and treatment complexity.

This study has several limitations. Most notably, the occurrence of thrombotic events in only four patients within the sample substantially limits the statistical power to analyze potential risk factors associated with thrombosis. Furthermore, the presence of comorbidities in some patients, especially those already receiving antithrombotic therapy, complicates the ability to infer a direct causal relationship between thromboembolic complications and COVID-19 infection. The retrospective nature of the study and the lack of data on the timing and dosage of concurrent medications introduce additional uncertainty. Therefore, the findings should be interpreted with caution and primarily regarded as hypothesis-generating.

## 5. Conclusion

In conclusion, while COVID-19 infection may be associated with an increased risk of thrombotic events even in patients with mild-to-moderate symptoms who are managed in outpatient settings, the frequency of such events appears to be considerably lower compared to hospitalized patients, particularly those in ICUs. Given the very limited number of thromboembolic events observed in this study, our findings should be interpreted with caution and regarded as preliminary. Nonetheless, primary care physicians might consider paying close attention to patients' comorbidities, hematological profiles, and medication use, especially in those with pre-existing hematological conditions or receiving antithrombotic therapy, as these factors could potentially be associated with increased thrombotic risk. More robust studies are needed to clarify these associations and inform outpatient risk management after COVID-19.

## Figures and Tables

**Table 1:** Demographic and other characteristics of patients.

	Total	Thrombosis	No thrombosis	*p* ^∗^
Gender, *n* (%)				> 0.05
Female	519 (54.0)	4 (100.0)	515 (53.8)	
Male	442 (46.0)	0	442 (46.2)	
Age, median (min–max)	41 (18–97)	58.5 (25–75)	41 (18–97)	> 0.05
Marital status, *n* (%)				> 0.05
Single	329 (34.0)	1 (25.0)	328 (34.3)	
Married	567 (58.6)	2 (50.0)	565 (59.0)	
Widow	65 (6.7)	1 (25.0)	64 (6.7)	
Education level				> 0.05
Illiterate	20 (2.1)	0	20 (2.1)	
Primary school	182 (18.8)	0	182 (19.0)	
Middle school	16 (1.7)	0	16 (1.7)	
High school	264 (27.3)	1 (25.0)	263 (27.5)	
College	329 (34.0)	1 (25.0)	328 (34.3)	
Degree	150 (15.5)	2 (50.0)	148 (15.5)	
Comorbidity, *n* (%)				> 0.05
Not present	636 (66.2)	1 (25.0)	635 (66.4)	
Present	325 (33.8)	3 (75.0)	322 (33.6)	
Hypertension	154 (16.0)	2 (50.0)	152 (15.9)	> 0.05
Diabetes mellitus	67 (7.0)	0	67 (7.0)	> 0.05
Hyperlipidemia	68 (7.1)	0	68 (7.1)	> 0.05
Asthma/COPD^1^	34 (3.5)	0	34 (3.6)	> 0.05
Coronary artery disease	42 (4.4)	1 (25.0)	41 (4.3)	> 0.05
Chronic heart failure	6 (0.6)	0	6 (0.6)	> 0.05
Atrial fibrillation	2 (0.2)	0	2 (0.2)	> 0.05
Other cardiac dysrhythmia	15 (1.6)	0	15 (1.6)	> 0.05
Mitral stenosis	1 (0.1)	0	1 (0.1)	> 0.05
Chronic venous insufficiency	3 (0.3)	0	3 (0.3)	> 0.05
Hematologic diseases	5 (0.5)	1 (25.0)	4 (0.4)	**< 0.05**
Endocrine diseases	57 (5.9)	0	57 (6.0)	> 0.05
Malignancies	15 (1.6)	0	15 (1.6)	> 0.05
Rheumatologic diseases	14 (1.5)	0	14 (1.5)	> 0.05
Kidney diseases	3 (0.3)	0	3 (0.3)	> 0.05
Cerebrovascular disease	4 (0.4)	0	4 (0.4)	
Drug utilization, *n* (%)				> 0.05
No	661 (68.4)	1 (25.0)	660 (69.0)	
Yes	300 (31.0)	3 (75.0)	297 (31.0)	
Medications (ATC^2^ codes),				
Drugs for acid-related disorders (A02)	41 (4.2)	1 (25.0)	40 (4.2)	> 0.05
Drugs used in diabetes (A10)	57 (5.9)	0	57 (6.0)	> 0.05
Antithrombotic agents (B01)	85 (8.8)	2 (50.0)	83 (8.7)	**< 0.05**
Cardiac therapy (C01)	12 (1.2)	0	12 (1.3)	> 0.05
Diuretics (C03)	9 (0.9)	0	9 (0.9)	> 0.05
Beta blocking agents (C07)	71 (7.3)	1 (25.0)	70 (7.3)	> 0.05
Calcium channel blockers (C08)	25 (2.6)	0	25 (2.6)	> 0.05
Agents acting on RAS^3^ (C09)	96 (9.9)	1 (25.0)	95 (9.9)	> 0.05
Lipid modifying agents (C10)	52 (5.4)	0	52 (5.4)	> 0.05
Endocrine drugs (H)	47 (4.9)	0	47 (4.9)	> 0.05
Antineoplastic and immunomodulating agents (L)	10 (1.0)	0	10 (1.0)	> 0.05
Musculoskeletal system (M)	7 (0.7)	0	7 (0.7)	> 0.05
Nervous system (N)	44 (4.6)	1 (25.0)	43 (4.5)	> 0.05
Drugs for obstructive airway diseases (R03)	24 (2.5)	0	24 (2.5)	> 0.05
Antihistamines for systemic use (R06)	7 (0.7)	0	7 (0.7)	> 0.05
Polypharmacy, *n* (%)				> 0.05
No	920 (95.1)	4 (100.0)	916 (95.7)	
Yes	41 (4.2)	0	41 (4.3)	
Total, *n* (%)	**961**	**4**	**957**	

*Note:* Bold values are statistically significant.

^∗^Fisher's exact test.

^1^Chronic obstructive pulmonary disease.

^2^Anatomical Therapeutic Chemical Classification System.

^3^Renin–angiotensin system.

**Table 2:** Effects of medications utilized in the last six months on thrombosis.

	Thrombosis			
	OR	%95 CI		*p* value^∗^
Medications (ATC^1^ codes)				
Drugs for acid-related disorders (A02)	36.7	1.5	900.4	**0.03**
Drugs used in diabetes (A10)	0.1	0.0	N/A	1
Antithrombotic agents (B01)	20.7	0.4	1039.2	0.13
Cardiac therapy (C01)	0.0	0.0	N/A	1
Diuretics (C03)	231.4	0.0	N/A	1
Beta blocking agents (C07)	0.6	0.0	23.3	0.80
Calcium channel blockers (C08)	0.0	0.0	N/A	1.00
Agents acting on RAS^2^ (C09)	0.9	0.0	36.5	0.97
Lipid modifying agents (C10)	0.3	0.0	N/A	1
Endocrine drugs (H)	0.0	0.0	N/A	1.00
Antineoplastic and immunomodulating agents (L)	0.0	0.0	N/A	1.00
Musculoskeletal system (M)	0.0	0.0	N/A	1.00
Nervous system (N)	3.6	0.2	80.1	0.42
Drugs for obstructive airway diseases (R03)	0.0	0.0	N/A	1.00
Antihistamines for systemic use (R06)	0.0	0.0	N/A	1.00
Covariates				
Age	1.0	0.9	1.1	0.89
Hypertension	4.9	0.1	376.7	0.48
Diabetes mellitus	0.0	0.0	N/A	1.00
Hyperlipidemia	0.0	0.0	N/A	1.00
Asthma/chronic obstructive pulmonary disease	0.0	0.0	N/A	1.00
Coronary artery disease	16.3	0.3	1009.7	0.19
Chronic heart failure	1.3	0.0	N/A	1
Atrial fibrillation	0.5	0.0	N/A	1
Other cardiac dysrhythmia	0.0	0.0	N/A	1.00
Mitral stenosis	2309.3	0.0	N/A	1
Chronic venous insufficiency	0.0	0.0	N/A	1.00
Hematologic diseases	132.6	2.8	6229.8	**0.01**
Endocrine diseases	0.0	0.0	N/A	1.00
Malignancies	0.0	0.0	N/A	1.00
Rheumatologic diseases	0.0	0.0	N/A	1.00
Kidney diseases	0.0	0.0	N/A	1
Cerebrovascular disease	252.2	0.0	N/A	1

*Note:* Bold values are statistically significant.

^∗^Logistic regression.

^1^Anatomical Therapeutic Chemical Classification System.

^2^Renin–angiotensin system.

**Table 3:** Details of thrombotic events of patients with thrombosis after COVID-19.

	Sex	Age	Comorbidity	Thrombotic event	Treatment of thrombotic event
Patient 1	F	25	No	DVT	Enoxaparin sodium, warfarin
Patient 2	F	47	Gastritis	Pulmonary embolism	Enoxaparin sodium
Patient 3	F	70	HT, polycythemia vera	DVT	Acetylsalicylic acid, intermittent pneumatic compression
Patient 4	F	75	CAD, HT	MI	Coronary artery stent

*Note:* HT: hypertension.

Abbreviations: CAD, coronary artery disease; DVT, deep vein thrombosis; MI, myocardial infarction.

## Data Availability

The data that support the findings of this study are available from the corresponding author upon reasonable request.
